# The landscape epidemiology of echinococcoses

**DOI:** 10.1186/s40249-016-0109-x

**Published:** 2016-02-19

**Authors:** Angela M. Cadavid Restrepo, Yu Rong Yang, Donald P. McManus, Darren J. Gray, Patrick Giraudoux, Tamsin S. Barnes, Gail M. Williams, Ricardo J. Soares Magalhães, Nicholas A. S. Hamm, Archie C. A. Clements

**Affiliations:** Research School of Population Health, The Australian National University, Canberra, New South Wales Australia; Ningxia Medical University, Yinchuan, Ningxia Hui Autonomous Region P. R. China; Molecular Parasitology Laboratory, QIMR Berghofer Medical Research Institute, Brisbane, Queensland Australia; Chrono-environment lab, UMR6249, University of Bourgogne Franche-Comté/CNRS, Besançon, France; Institut Universitaire de France, Paris, France; The University of Queensland, School of Veterinary Science, Gatton, Queensland Australia; The University of Queensland, Queensland Alliance for Agriculture and Food Innovation, Gatton, Queensland Australia; School of Public Health, The University of Queensland, Brisbane, Queensland Australia; Child Health Research Centre, The University of Queensland, Brisbane, Queensland Australia; Faculty of Geo-Information Science and Earth Observation (ITC), University of Twente, Enschede, The Netherlands

**Keywords:** Landscape epidemiology, Helminth infection, Human echinococcosis, *Echinococcus* spp, Environmental change, Geographic information systems, Remote sensing, Geostatistics

## Abstract

Echinococcoses are parasitic diseases of major public health importance globally. Human infection results in chronic disease with poor prognosis and serious medical, social and economic consequences for vulnerable populations. According to recent estimates, the geographical distribution of *Echinococcus* spp. infections is expanding and becoming an emerging and re-emerging problem in several regions of the world. Echinococcosis endemicity is geographically heterogeneous and over time it may be affected by global environmental change. Therefore, landscape epidemiology offers a unique opportunity to quantify and predict the ecological risk of infection at multiple spatial and temporal scales. Here, we review the most relevant environmental sources of spatial variation in human echinococcosis risk, and describe the potential applications of landscape epidemiological studies to characterise the current patterns of parasite transmission across natural and human-altered landscapes. We advocate future work promoting the use of this approach as a support tool for decision-making that facilitates the design, implementation and monitoring of spatially targeted interventions to reduce the burden of human echinococcoses in disease-endemic areas.

**Electronic supplementary material:**

The online version of this article (doi:10.1186/s40249-016-0109-x) contains supplementary material, which is available to authorized users.

## Multilingual abstracts

Please see Additional file 1 for translations of the abstract into the six official working languages of the United Nations.

## Introduction

Landscape epidemiology is the study of the spatial variation in disease risk, in strong connexion with landscape characteristics and relevant environmental factors that influence the dynamics and distribution of host, vector and pathogen populations. The fundamental concepts of landscape epidemiology were formalised and introduced by the Russian parasitologist, Pavlovsky, in 1966 [1]. According to Pavlovsky, landscape epidemiology is based on three observations: first, diseases tend to be limited geographically; second, the spatial variation in the distribution of a disease is determined by variations of physical and/or biological conditions that support a pathogen, its vectors and reservoirs; and third, the contemporaneous and future risk of a disease can be predicted if those conditions are mapped [2]. This conceptual framework has been developed and extended progressively to integrate concepts and approaches from multidisciplinary studies, including landscape ecology, for a better understanding of the complex composition of the landscape and its relationship with the transmission processes and geographical distribution of a disease [3–5]. The current principles of landscape epidemiology have been recently summarised in a set of propositions outlined by Lambin and colleagues (Table 1) [6].

**Table 1:** The 10 principles of landscape epidemiology proposed by Lambin and colleagues

Principle	Description
1	Landscape attributes may influence the level of transmission of an infection
2	Spatial variations in disease risk depend not only on the presence and area of critical habitats but also on their spatial configuration
3	Disease risk depends on the connectivity of habitats for vectors and hosts
4	The landscape is a proxy for specific associations of reservoir hosts and vectors linked with the emergence of multi-host disease
5	To understand ecological factors influencing spatial variations of disease risk, one needs to take into account the pathways of pathogen transmission between vectors, hosts, and the physical environment
6	The emergence and distribution of infection through time and space is controlled by different factors acting at multiple scales
7	Landscape and meteorological factors control not just the emergence but also the spatial concentration and spatial diffusion of infection risk
8	Spatial variation in disease risk depends not only on land cover but also on land use, via the probability of contact between, on one hand, human hosts and, on the other hand, infectious vectors, animal hosts or their infected habitats
9	The relationship between land use and the probability of contact between vectors and animal hosts and human hosts is influenced by land ownership
10	Human behaviour is a crucial controlling factor of vector-human contacts, and of infection.

Most modern landscape epidemiological studies use Earth observation (EO) to obtain remotely sensed (RS) and in situ data about the environment [5]. Geographic information systems (GIS) are used to capture, store, analyse and display geo-referenced data that may be exported to various analytical and statistical platforms [5]. The integrated use of these technologies and the application of spatiotemporal statistics allow investigators to explore in detail the landscape patterns that influence the transmission dynamics of an infectious disease at different spatiotemporal scales. EO, GIS and the use of innovative analytical methods also provide the opportunity to visualise and predict the geographical variations in disease risk in response to shifting environmental patterns [7, 8]. In this way, landscape epidemiology may offer a feasible and acceptable framework to reduce disease burdens by allowing a more precise estimation of populations at high risk and the identification of priority areas where allocation of disease control resources is most required [9].

To date, landscape epidemiology has been mainly applied to examine associations between the environment and the transmission dynamics of mosquito-borne diseases such as malaria, dengue, leishmaniasis, filariasis and trypanosomiasis [10–13]. However, with the advent of global environmental change, there has been an increasing interest in conducting studies centred on the understanding of the landscape epidemiological aspects of non-mosquito-borne helminth infections, such as schistosomiasis [14–16]. This approach has been successful in providing valuable information to enhance the implementation of strategies for surveillance, control and elimination of helminth infections in various settings [17–19].

Echinococcoses are zoonotic parasitic diseases caused by larval stages of taeniid cestodes of the genus *Echinococcus*. Currently, there are nine recognised species within the genus and six of these species cause infection in humans, *E. granulosus*, *E. multilocularis*, *E. canadensis*, *E. ortleppi*, *E. vogeli* and *E. oligarthrus* [20, 21]. Among them, *E. granulosus*, the main aetiological agent of cystic echinococcosis (CE), and *E. multilocularis*, the causative agent of alveolar echinococcosis (AE), are the species of major public health importance globally [22]. Both have a wide geographic distribution and cause severe disease in humans that can be fatal if left untreated [23–25]. The other two less common forms of human infection are polycystic echinococcosis and unicystic echinococcosis caused by *Echinococcus* species restricted to Central and South America [25].

There are approximately 200,000 new cases of human CE or AE cases diagnosed every year and a total of 2–3 million people infected worldwide [26, 27]. According to the *Office International des Epizooties* databases and published case reports, the estimated human burden of CE measured in terms of Disability-Adjusted Life Years (DALYs) lost is 285,407. When underreporting in accounted for, the global burden of this form of infection exceeds 1 million DALYS, which results in an annual estimated cost of $760 million [26]. Global estimates of AE suggest that there are approximately 18,235 people infected every year and a total of 0.3–0.5 million AE cases diagnosed worldwide. Most of the disease burden of AE is focused on Western China and results in the loss of 666,434 DALYs per annum [28]. Although these reports may be underestimates due to challenges with the early detection of the diseases and lack of mandatory reporting in most countries, it is apparent that the burden of echinococcoses has increased in recent years and human infection is becoming an emerging or re-emerging problem in several regions in the world [29–36]. Consequently, landscape epidemiological approaches have been incorporated progressively into echinococcosis research to identify the environmental mechanisms underlying the variation in disease risk and the most plausible drivers of parasite dispersion [37–43].

This review aims to describe the potential applications of landscape epidemiological studies to establish, quantify and predict the geographical distribution of human echinococcoses and as a decision-making tool to enhance the implementation of spatially targeted interventions against the disease. First, the review describes important epidemiological features of the parasite and discusses some of the most relevant biophysical environmental factors that may affect the distribution of echinococcosis risk at different spatial scales. Next, the review describes how landscape epidemiology may use geospatial resources and techniques to improve the understanding of the transmission dynamics of *Echinococcus* spp., and facilitate the strategic allocation of resources for interventions to the appropriate geographic locations. Finally, challenges and gaps in the current evidence are identified and research priorities to support the surveillance and control of human echinococcoses are proposed.

### Search strategy

A search was conducted of literature including all relevant articles that were published until September 2015, identified from Medline, Google Scholar, PubMED and Web of Knowledge. The key terms used in the search strategy included one word and/or phrase from each of the following three categories: first, terms related to the disease, including zoonoses, parasitic disease, helminth infections, human hydatidosis, hydatid cyst, cystic echinococcosis and alveolar echinococcosis; second, terms related to risk factors for parasite transmission, including environmental influences, climate change, anthropogenic environmental factors, and landscape; and third, terms related to the analytical approach, including landscape epidemiology, risk mapping, geographic information systems, remote sensing, Bayesian analysis, geostatistics and geospatial techniques and/or methods. Additionally, secondary searches were conducted in reference lists of peer-reviewed studies. The language of the literature was restricted to English.

### Environmental determinants of the multi-spatial variation in human echinococcosis risk

*Echinococcus* spp. have complex domestic and sylvatic life cycles that involve a wide range of intermediate and definitive hosts. Therefore, echinococcosis transmission can take place in different landscape types in which a variety of physical and biological factors combine to determine the transmission intensity of the parasite [25]. Although these factors remain poorly understood, it is apparent that the environment plays an important role in the life cycle of *Echinococcus* spp. Climate and landscape structure influence particularly human behaviour, animal population dynamics, spatial and temporal overlap of intermediate and definitive hosts and the survival of the parasite eggs [41, 44–47]. Humans, who become infected by ingesting the parasite eggs directly through contact with definitive hosts or indirectly from a contaminated environment, are regarded as accidental intermediate hosts who do not usually contribute to the developmental cycle of the parasite. However, reports from hyperendemic areas in north-western Kenya indicate that humans may act as intermediate hosts in the life cycle of the parasite under unique circumstances. The close human-dog relationship and the absence of burial customs among the Turkana people in this region, seem to have made possible the transmission of *E. granulosus* from tribesmen to dogs or wild carnivores which are able to access and scavenge potentially infected human remains [48]. Comprehensive reviews of the parasite life cycle, environmental factors influencing parasite transmission, clinical manifestations, diagnosis and management of the disease are available [22, 25, 44, 49].

There is an important spatial dimension in the relationship between the risk of echinococcosis infection and environmental factors that influence both the distribution of the hosts and the rate of development of the parasite [45, 50]. Despite the extent of epidemiological variations within the genus *Echinococcus*, a general framework may be used to describe factors driving the transmission of the parasite at the continental, sub-continental and local spatial scales. At the continental level, echinococcosis risk may be related to the philogeography (biogeography) of animal communities and to variations in climatic conditions that control the presence/absence of host species within a particular landscape type [41]. At the sub-continental level, the spatiotemporal patterns of echinococcosis risk depend upon animal population dynamics, predator–prey interactions and parasite free living stage survival. Thus, at this spatial scale, the infection is likely to be associated with landscape characteristics, such as composition (variety and abundance of patch types in the landscape), and configuration (spatial arrangement and complexity of patches present in the landscape) that together with climatic factors determine the seasonal and interannual variation in population density of the hosts, parasite free stage survival, and subsequently the geographical distribution of *Echinococcus* spp. [50].

To date, most studies conducted at sub-continental spatial scale have focused on describing the role of landscape composition in determining the risk of infection with *E. multilocularis* in wildlife [41–43, 51–55]. In eastern France, high population densities of *Microtus arvalis* and *Arvicola terrestris*, vole species that are key intermediate hosts for *E. multilocularis*, were identified in areas where ploughed fields were converted into permanent grassland as a result of the local specialisation in milk production in the 1960s and 1970s [41, 56]. In addition, significant positive relationships between percentage of area covered by grassland and *E. multilocularis* infection in humans and foxes have also been reported in the same region [39, 41, 57]. Studies conducted in Zhang County, Gansu Province, China, indicated that the transmission of *E. multilocularis* may be related to the transient augmentation of grassland/shrubland following a period of deforestation. In this hyperendemic area for AE, land cover change favoured the creation of optimal peri-domestic habitats for AE intermediate host species, and the development of a peri-domestic cycles involving dogs [41, 58]. In AE-endemic areas from the north-western part of Sichuan Province on the Tibetan Plateau, private partial fencing has been common among Tibetan pastoral communities since the 1980s. This practice allows the creation of private grazing areas to support livestock during the winter period and early spring. Although fenced pasture has reduced grazing pressure in private areas, it has also exacerbated overgrazing in common lands and has improved suitability of habitats for various rodent species that are vulnerable to the parasite. As a result, the risk of AE has also increased in the region [54, 59, 60]. By contrast, in northern Japan, grey-sided voles form large populations in dense bamboo undergrowth of forest. Since this land cover is natural vegetation, AE prevalence in this part of the country appears to be not related to anthropogenic landscape changes [61, 62].

Despite compelling evidence supports the association of the environment with the spatial variation of *E. multilocularis* infection in sylvatic systems [41, 43, 51–55, 63], little is known about the host-environment interactions that take place at sub-continental levels to regulate the transmission of *E. granulosus* in domestic settings, where dogs are identified as typical definitive hosts, and sheep and other ungulates, as intermediate hosts [25]. Livestock like any other animal system can be influenced by climate and landscape resources that shape animal feeding behaviour, growing rates, reproductive efficiency and immunological mechanisms that protect against pathological and non-pathological stressors [64]. Heat stress, particularly, declines feeding intake, conception rates and the immune response to infectious diseases in sheep and cattle [64, 65]. Therefore, climate change and landscape transformation, together with high level of environmental contamination with parasite eggs have the potential to affect parasite transmission intensity not only in wildlife but also in urban settings, and consequently increase the risk of human CE. Reports from abattoir meat inspections suggested seasonal variations in the prevalence of *E. granulosus* infection in Iran and Saudi Arabia [66, 67]. Additionally, high altitudes and annual rainfall were associated with high infection rates of CE in livestock from hyperendemic regions for this infection in north-central Chile and Ethiopia [68, 69]. The observations from these countries were explained by factors such as sources of slaughtered animals, different animal age-structures among seasons, changes in agricultural management practices and environmental factors. The geographical location of livestock farms and the animal spatial structure also appeared to have an important effect on the prevalence of CE in the Campania region of southern Italy. Using geo-referenced data, a survey conducted in this region suggested that the significantly higher prevalence of CE on cattle farms compared to water buffalo farms was associated with their closer distance to potentially infected sheep [70].

At local or community spatial scales, microclimate is one of the most significant factors underlying the variation in the risk of echinococcosis infection [46, 47]. Temperature and moisture/humidity, particularly, are major determinants of the survival and longevity of the parasite eggs in the external environment [46, 47]. Although the optimal temperature range for egg survival has been estimated to be between 0 and 10 °C, the tolerance of the eggs to external environmental conditions varies between parasite species and strains [46, 47]. For *E. multilocularis* eggs, temperatures of 4 and of −18 °C were found to be well tolerated, with survival times of 478 and 240 days, respectively [46]. In addition, a recent study showed that *E. multilocularis* eggs are more resistant to heat if suspended in water compared to eggs exposed to heat on a filter paper at 70 % relative humidity. Eggs suspended in water can remain infectious for up to 120 min if expose to temperatures of 65 °C [71]. In vivo studies also revealed that the eggs of *E. granulosus* remain viable and infective after 41 months of exposure to an inferior arid climate, which is characterised by large thermal amplitude (from −3 to 37 °C), with warm summers, cold winters and low precipitation (under 300 mm/year) [47].

At the local level, human behavioural changes, driven in large part by population growth and economic and technological development, have been associated with the creation of novel interactions between humans, domestic animals and wildlife [72]. This new human-environment interplay also appears to be altering human exposure to *Echinococcus* spp. by facilitating the establishment and introduction of competent intermediate and definitive hosts in the life cycle of the parasite [73, 74]. Foxes, the primary definitive host of *E. multilocularis*, take advantage of the most accessible and abundant resources of water and food. Therefore, the reported movement of foxes towards urban areas, where the transformed landscapes provide optimal conditions for surges of small mammal species, have explained the observed higher circulation of the parasite within local urban landscapes [73]. In addition, the role of dogs in semi-domestic life cycles of *E. multilocularis* appears to be the result of human-related activities in certain communities where dog ownership and close association between humans and dogs were identified as significant predictors of human AE risk. [75–77]. Similarly, reports have revealed that urban coyotes are currently playing a key role in the maintenance of the life-cycle of *E. multilocularis* within North American urban settings [78].

**Fig. 1:**
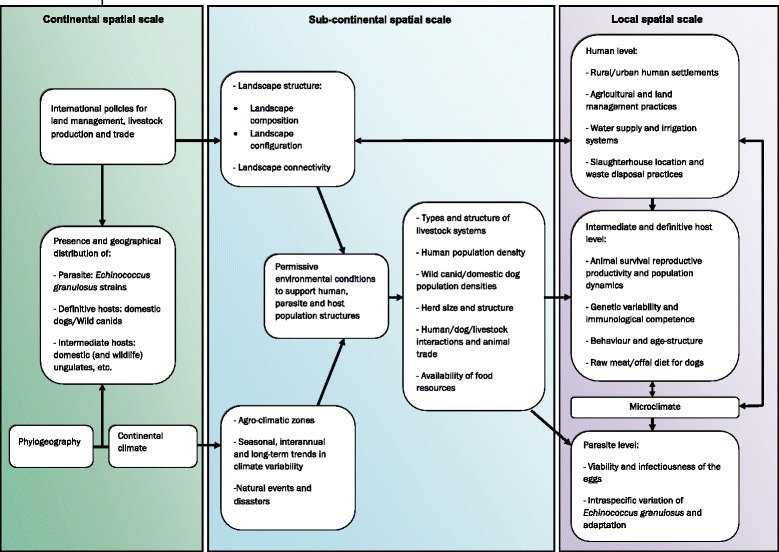
Conceptual diagram of the environmental factors influencing the transmission dynamics of *Echinococcus granulosus* at different spatial scales

**Fig. 2:**
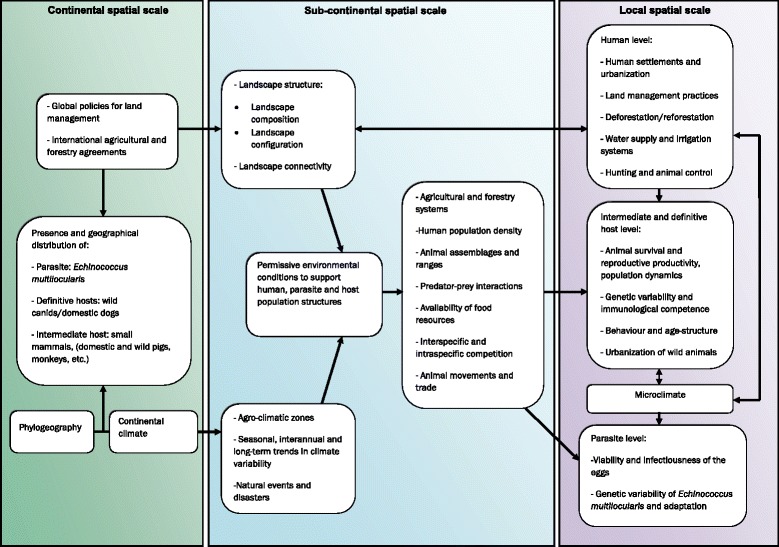
Conceptual diagram of the environmental factors influencing the transmission dynamics of *Echinococcus multilocularis* at different spatial scales

Genetic factors and immunological interactions between the parasite and hosts are also associated with echinococcosis risk at local and community levels. These factors affect the development of the adult parasite in the intestine of definitive hosts and determine the time course of the production and viability of the eggs [79]. Genetic and immunological factors also govern differences in the reproductive potential of the hosts and influence the susceptibility/resistance of humans and animals to the infection [80]. Patients with impaired immune response appear to have increased susceptibility to *E. granulosus* and *E. multilocularis* infections, and are more prone to develop severe disease [81–83]. Similarly, an increase risk of infection with *E. multilocularis* has been observed in experimental immunosuppressed animals [80]. Figures 1 and 2 show a conceptual diagram of the environmental factors influencing the transmission dynamics of *E. granulosus and E. multilocularis*, respectively, at different spatial scales.

### The use of landscape epidemiological approaches to understand the transmission dynamics of *Echinococcus* spp.

The inherently multi-scale nature of the life cycle of *Echinococcus* spp. has represented a challenge to comprehensively understand the mechanisms that govern parasite transmission and the subsequent variation in disease risk [79, 84]. However, over the past decade, advances in EO, that have led to the increased availability of high-quality environmental data, and developments in GIS and methods for spatial analysis have improved the ability of investigators to explore and predict the spatiotemporal dynamics of echinococcosis infections.

Much progress has been made in the use of geospatial technologies to map the prevalence of infection with *Echinococcus* spp. and identify space-time clusters of human disease in various settings [58, 70, 85–88]. With global environmental change, there has been a growing interest in determining the role of climatic factors and the process of landscape transformation in the recent observed patterns of parasite transmission. Thus, deforestation, grazing practices, climate variability and direct or indirect control of intermediate and definitive hosts are currently being studied as potential environmental factors that have favoured the persistence and geographical expansion of the parasite [41, 43, 61, 75].

Landscape epidemiology uses a wide variety of data and statistical techniques [5]. Accurate data, both in space and time, are required to develop statistical models that describe the complex associations between the environment and the transmission of the parasite [89]. Data collected at a specific geographic location can be geo-referenced using spatial coordinates, such as those obtained from global positioning systems (GPS). By contrast, data collected from a defined spatial region, such as clinical surveillance data for an administrative area, are geo-referenced by specifying the administrative boundaries, with some associated limitations for subsequent spatial analysis [89]. Because reporting of echinococcosis infections is not mandatory in most countries, epidemiological data are usually fragmented and scarce. In most endemic areas, human cases are primarily identified through clinical case reports, hospital records or mass screening surveys that usually combine questionnaires based-interviews, abdominal ultrasound and specific serology tests [90–93]. These initiatives have resulted in a valuable source of geospatial data for the estimation of echinococcosis risk at local and regional spatial scales, and at certain points in time in several endemic regions. However, these represent inefficient measures that are difficult to sustain in the long term [43]. The European Echinococcosis Registry (EurEchinoReg) Project was the first attempt to establish a continent-wide database for echinococcosis, with the aim being to estimate the impact of AE in western and central Europe. However, the routine collection of data by individual countries has been heterogeneous in terms of completeness and reliability across regions [94]. Since the beginning of the project, Austria, France, Germany and Switzerland are among the few countries that have maintained population-based human AE data registries that can be used to analyse patterns of this form of disease at various spatial scales [94–96].

In addition to data on human echinococcosis cases, data on environmental factors and survey data to determine the presence of echinococcosis host species and their infection status may also be combined in landscape epidemiological studies [45]. Although infection in definitive and intermediate hosts are key indicators of the presence of the parasite in the environment, the identification of infected animals does not directly reflect transmission pressures of *Echinococcus* spp. to human populations. Nevertheless, it can be assumed that environmental processes that support variation in host population densities are also likely to influence the risk of human infection [31, 41]. Sources of EO for environmental data include satellite remote sensing and spatially distributed in situ sensors, such as meteorological stations [97]. EO and its derived products provide extensive coverage of vast areas of the earth at periodic intervals. In the case of in situ data, interpolation methods can be applied to obtain data for those locations where there are no meteorological observations locally available [98, 99]. Currently, a wide range of high-quality environmental datasets are freely available and can be used to identify continental, sub-continental or local environmental variability [97]. The International Union for Conservation of Nature has also created databases for mapping the distribution of animal species, including most definitive and intermediate hosts of *Echinococcus* spp. [100]. The environmental variables most commonly used in echinococcosis research include altitude, temperature, precipitation, land cover, land use, vegetation indices and geographical distribution of the hosts [44, 75].

The characterisation and prediction of echinococcosis risk using landscape epidemiology can be achieved by using geospatial resources and spatial analysis methods that allow visualisation, exploration and modelling of multi-source geo-referenced data. Among them, GIS mapping and cluster detection techniques are useful tools that have been widely applied in echinococcosis research to prioritise areas for further studies and plan preventive and control interventions [70, 95, 101–103]. In general, these methods have indicated that echinococcosis infections have a focal spatial distribution, with defined areas at high risk for parasite transmission between definitive and intermediate hosts, in which the prevalence or incidence of human disease may be higher than in surrounding areas. Examples include studies undertaken in France, Japan and China, countries heavily affected by AE. In these countries, the evidence has suggested that the number of human cases of AE is a nested hierarchy of spatial aggregates in the eastern part of France. Aggregative distribution has also been shown in the northern island of Hokkaido, Japan, and in provinces located in the central and western part of China, where the Qinghai-Tibetan plateau has been identified as the geographic area with the highest rates of human AE recorded globally [41, 104, 105]. Similarly, epidemiological studies in north-western China revealed much higher prevalence of CE among local communities from the Tibet Autonomous Region, Xinjiang Uygur Autonomous Region, Ningxia Hui Autonomous Region (NHAR), and Sichuan and Qinghai Provinces [106]. Demographic, socio-economic and human behavioural factors are also variables that have been commonly explored as potential factors interacting with the environment to determine the heterogeneous spatial distribution of echinococcoses in several endemic regions. The Buddhist doctrine among pastoral communities that allows old livestock to die naturally, coupled with the practice of unrestricted disposal of animal viscera and the presence of free ranging dogs have been identified as factors influencing the high prevalence of human CE in Tibetan communities in China [106]. Significant difference in prevalence rates of human infection has also been observed between males and females. Women are more likely to be exposed to *E. granulosus* and *E. multilocularis* as a result of their daily family activities such as feeding dogs, herding livestock and collecting yak dung for fuel [85, 107, 108]. Additional risk factors found to be related to high risk of exposure to both parasite species include dog ownership, poor hygienic practices, low income and limited education. In contrast, the use of tap water has been identified as a factor that can protect against the disease [85, 93, 101, 107–109].

As a result of the apparently expanding geographical distribution of *Echinococcus* spp. [29–35], particular emphasis has recently been placed on the implementation of landscape epidemiological approaches that use spatial statistical techniques to identify environmental conditions that may be affecting the habitat suitability for sustaining the sylvatic life cycle of the parasite [42, 43, 53, 75, 110]. Spatial statistics are statistical methods that can be applied to explore geographically referenced data and investigate associations between the observed number of human cases and the most plausible factors that underlie the transmission dynamics of the parasite. On the basis of the information provided by this approach, traditional or spatially explicit statistical models can also be constructed to predict the spatial distribution of disease based on environmental variables. Of note, the statistical methods applied in epidemiology that fail to account for spatial autocorrelation in the variables used to model and predict disease risk, may possibly lead to erroneous statistical inference [111]. Thus, spatially explicit models that incorporate information on spatial autocorrelation, obtained using Bayesian methods are increasingly incorporated in landscape epidemiological research. Bayesian methods are sufficiently flexible to allow the development of complex hierarchical spatio-temporal models that quantify uncertainty in the analysis of disease risk by assuming that parameter values, including spatial predictions, vary as random quantities [112]. Predictive risk maps of echinococcoses that account for uncertainty estimates can be essential to inform decision-makers about the uncertainty and implications of the interventions against these infections [14, 113]. A Bayesian statistical framework was used in Xiji County, NHAR, China, for risk mapping and transmission modelling of human AE [43]. The study indicated that the landscape characteristics favouring *E. multilocularis* transmission in Xiji County differed from the previous observations in Zhang County located in the neighbouring Gansu Province. While grassland around villages did not correlate with the prevalence of human AE, abundance of degraded lowland pastures was associated with higher prevalence of the disease in Xiji County. From the results, it was possible to infer that *E. multilocularis* can sustain transmission through a diversity of host communities in China [43]. A similar Bayesian approach was carried out in Tibetan plateau communities, which led to confirm and predict human disease hotspots over a 200,000 km^2^ region and showed that human AE risk was better predicted from landscape features [75].

### Applications to surveillance, prevention and control programmes

Landscape epidemiology has been applied progressively in echinococcosis research, particularly AE research, in order to identify the environmental determinants of echinococcosis risk. However, there is still limited guidance on the practical implementation of this approach to improve echinococcosis surveillance and maximise the impact of prevention and control efforts.

Most of the evidence in the use of landscape epidemiology to support the effective implementation of interventions against infectious diseases has been obtained from studies of mosquito-borne diseases [10, 11, 13], and non-mosquito-borne helminth infections, particularly schistosomiasis and soil-transmitted helminthiases [17–19]. At the global scale, atlases have been developed that may potentially guide international priority setting for investments in disease control and elimination [114–116].

In the context of echinococcosis surveillance and control, mass screening surveys of echinococcoses have provided valuable data to help reduce the medical, social and economic burden of the infection by ensuring early detection and prompt treatment of human cases. However, this measure may be inefficient and resource intensive if implemented in areas of low prevalence of the disease. Echinococcoses affect particularly remote pastoral communities with low socioeconomic development that may have limited access to health care [108, 117]. Therefore, landscape epidemiological studies have the potential to assist local and national initiatives against echinococcoses such as the one launched by the Chinese government to reduce the impact of these infections in 217 endemic counties in western China [23, 118]. Such studies generate both quantitative evidence and visual representation of the geographical distribution of these diseases and allow a more precise estimation of populations at high risk. Updated maps of echinococcoses and accurate information about individuals and households at high risk may allow decision makers to optimally target resources and interventions for prevention and control.

In China, particularly, the current measures adopted against echinococcoses include community-based epidemiological surveys, patient treatment and monitoring, health education campaigns, and regular antihelmintic treatment for dog deworming [23, 118]. Under the strategic and operational context of these interventions and other potential strategies that may help reduce the burden of these infections in endemic regions, landscape epidemiological approaches represent a cost-effective measure not only to prioritise geographical areas at high risk, but also to identify the type of parasite control activity that is most required in specific locations. Deworming of wild foxes using baits with antihelmintic treatment is being established in some countries as a preventive technique against environmental contamination with *E. multilocularis* eggs [119–121]. In order to improve the cost-benefit performance of these efforts, spatial models were developed in Hokkaido, Japan, and in eastern France to identify the environmental factors that determine the most suitable micro-habitats for delivering the baits. The outcomes of these studies suggested that baiting programmes should be adapted to the local environmental characteristics of domestic and urban settings [119, 122].

Many of the relationships that have been explored in the studies outlined above have provided compelling evidence about the environmental conditions that together with socio-economic and demographic factors support the transmission of *Echinococcus spp*. in endemic regions. However, they fall short of allowing resource managers and policy makers to understand and anticipate the real impact of the infections, and the economic and medical implications of their decisions. Thus, approaches that incorporate the use of geospatial resources and spatial analysis to identify environmental drivers of echinococcoses can be applied as decision-making tools for the design of effective surveillance and response systems. In this way, landscape epidemiological studies may help monitor and predict parasite transmission based on changing environmental factors, and in response to the implementation of interventions for disease control. Most importantly, these approaches have the potential to guide echinococcosis control programmes in those regions with limited availability of surveillance data on echinococcoses [123].

The understanding of the landscape epidemiological aspects of echinococcoses may also provide scientific evidence that can be used to support environmental policy-making and landscape planning processes in hyperendemic areas for these diseases. Thus landscape epidemiology may also prove useful to promote environmentally-based strategies that have minimal impact on the transmission dynamics of the different *Echinococcus* spp. This is particularly relevant in regions where climate variability and landscape transformation may be facilitating the transmission of the parasite.

Previous studies conducted in echinococcosis-endemic regions have provided valuable insight into the landscape processes underlying the transmission of *E. multilocularis* at various spatial scales. However, most of these analyses involved environmental data collected at a single point in time and did not capture major environmental changes over time [50]. Because human echinococcoses may be the result of cumulative events that occurred over many years prior to the detection of the disease, the use of multi-temporal Earth observation datasets to identify environmental change will be necessary in order to conduct a meaningful landscape epidemiological analysis of the forms of human echinococcosis infections. Therefore, we advocate future research that incorporates time-series analyses of environmental data for the identification of the long-term trends in climatic and landscape conditions that may be facilitating the persistence and spread of *Echinococcus* spp. across heterogeneous landscapes.

Despite the potential applications of landscape epidemiology in echinococcosis research, it is evident that work is still necessary to address the limited availability of human echinococcosis data. Thus, further advances are required to improve long-term and multi-scale monitoring of these infections. We believe that the design and implementation of systematic and standardised protocols for the diagnosis, collection and recording of human cases may help to better estimate and monitor the prevalence of these infections in endemic areas, and also to increase awareness among all actors involved in the control of these infections. In addition, we also recommend the development of national and sub-national data collection systems to record all confirmed cases of echinococcoses identified through mass screening surveys or clinical and laboratory reports. Systematic surveillance systems may be used as efficient, reliable and secure data sources for the implementation of clinical and landscape epidemiological studies. Because echinococcoses are complex diseases that involve animal and human hosts, as well as ecological and environmental factors, integrated multisectoral efforts are clearly required to monitor the interactions between the landscape and parasite, hosts and human diseases. The availability of data on annual infection rates in humans, definitive and intermediate hosts in hyperendemic areas combined with annual averages of climate data and land cover change may be particularly useful to improve cost-effectiveness of small-scale campaigns and reduce local risk. These data are essential to establish pre-intervention baseline, monitor the efficacy of interventions and inform the strategic planning of future control measures.

Factors that need to be considered for the routinely implementation of these approaches include the availability of resources for collecting, processing, and modelling geospatial data at various spatial scales, training of personnel on the use of these technologies and the proper interpretation of results, and the continuous availability of high quality environmental data. It should be emphasized that the allocation of resources for the implementation of these novel techniques should not come at the cost of preventive and control efforts against the infections. Co-endemicity and polyparasitism are common in several regions of the world [124]. Therefore, initiatives to combine control strategies against human echinococcoses with other zoonotic diseases could potentially help to optimise resources, ensure sustainability of interventions and improve awareness among local people [124]. Major integrated programmes to map the distribution and enhance control strategies against some neglected tropical diseases such as onchocerciasis, lymphatic filariasis, soil-transmitted helminthiases and schistosomiasis are currently being implemented successfully in various regions [125]. In the context of echinococcoses, integrated dog control/deworming and health promotion may be proposed as a cost-effective measure to reduce the impact of these infections in highly endemic areas.

## Conclusion

This review demonstrates the potential of landscape epidemiology to explore the complex life cycle of *Echinococcus* spp. that involves time-dependent interactions of multiple definitive and intermediate hosts at different spatial scales. Landscape epidemiology has also proven helpful in characterising the geographical distribution of human AE risk and in determining the association between the geographical patterns of infection and environmental factors. Therefore, the implementation of this approach together with the recent advances in geospatial technologies and spatial analysis techniques provide a unique opportunity to explore the causes of persistence, emergence and re-emergence of some parasite species in several regions, and a better guidance for the design, implementation and monitoring of preventive and control interventions.

## Additional file

Additional file 1:
**Multilingual abstracts in the six official working languages of the United Nations.** (PDF 336 kb)

## Abbreviations

EO: Earth observation
GIS: geographic information systems
RS: remote sensing
CE: cystic echinococcosis
AE: alveolar echinococcosis
DALYs: disability-adjusted life years
GPS: global positioning systems
EURECHINREG: the European Echinococcosis Registry
NHAR: Ningxia Hui Autonomous Region

## References

[1] Ostfeld RS, Glass GE, Keesing F (2005). Spatial epidemiology: an emerging (or re-emerging) discipline. Trends Ecol Evol.

[2] Pavlovskii EN, Levine ND (1966). Natural nidality of transmissible diseases, with special reference to the landscape epidemiology of zooanthroponoses.

[3] Giraudoux P, Raoul F, Pleydell D, Craig PS (2008). Multidisciplinary studies, systems approaches and parasite eco-epidemiology: something old, something new. Parasite.

[4] Reisen WK (2010). Landscape epidemiology of vector-borne diseases. Annu Rev Entomol.

[5] Meentemeyer RK, Haas SE, Václavík T (2012). Landscape epidemiology of emerging infectious diseases in natural and human-altered ecosystems. Annu Rev Phytopathol.

[6] Lambin EF, Tran A, Vanwambeke SO, Linard C, Soti V (2010). Pathogenic landscapes: interactions between land, people, disease vectors, and their animal hosts. Int J Health Geogr.

[7] Robinson T (2000). Spatial statistics and geographical information systems in epidemiology and public health. Adv Parasitol.

[8] Hay SI, Randolph SE, Rogers DJ (2000). Remote sensing and geographical information systems in epidemiology.

[9] Eisen L, Eisen RJ (2011). Using geographic information systems and decision support systems for the prediction, prevention, and control of vector-borne diseases. Annu Rev Entomol.

[10] Parham PE, Michael E (2010). Modelling climate change and malaria transmission. Adv Exp Med Biol.

[11] Vanwambeke SO, Van Benthem BH, Khantikul N, Burghoorn-Maas C, Panart K, Oskam L (2006). Multi-level analyses of spatial and temporal determinants for dengue infection. Int J Health Geogr.

[12] Bavia M, Carneiro D, da Costa Gurgel H, Filho CM, Barbosa MR (2005). Remote sensing and geographic information systems and risk of American visceral leishmaniasis in Bahia. Brazil Parassitologia.

[13] Wardrop NA, Fèvre EM, Atkinson PM, Kakembo AS, Welburn SC (2012). An exploratory GIS-based method to identify and characterise landscapes with an elevated epidemiological risk of Rhodesian human African trypanosomiasis. BMC Infect Dis.

[14] Brooker S (2007). Spatial epidemiology of human schistosomiasis in Africa: risk models, transmission dynamics and control. Trans R Soc Trop Med Hyg.

[15] Clements AC, Garba A, Sacko M, Touré S, Dembelé R, Landouré A (2008). Mapping the probability of schistosomiasis and associated uncertainty, West Africa. Emerg Infect Dis.

[16] Soares Magalhães RJ, Salamat MS, Leonardo L, Gray DJ, Carabin H, Halton K (2014). Geographical distribution of human Schistosoma japonicum infection in The Philippines: tools to support disease control and further elimination. Int J Parasitol.

[17] Clements AC, Lwambo NJ, Blair L, Nyandindi U, Kaatano G, Kinung’hi S (2006). Bayesian spatial analysis and disease mapping: tools to enhance planning and implementation of a schistosomiasis control programme in Tanzania. Tropical Med Int Health.

[18] Brooker S, Clements AC, Bundy DA (2006). Global epidemiology, ecology and control of soil-transmitted helminth infections. Adv Parasitol.

[19] Magalhães RJS, Clements AC (2011). Mapping the risk of anaemia in preschool-age children: the contribution of malnutrition, malaria, and helminth infections in West Africa. PLoS Med.

[20] Rojas CAA, Romig T, Lightowlers MW (2014). Echinococcus granulosus sensu lato genotypes infecting humans–review of current knowledge. Int J Parasitol.

[21] McManus D (2013). Current status of the genetics and molecular taxonomy of Echinococcus species. Parasitology.

[22] McManus DP, Gray DJ, Zhang W, Yang Y (2012). Diagnosis, treatment, and management of echinococcosis. BMJ.

[23] McManus DP, Zhang W, Li J, Bartley PB (2003). Echinococcosis. Lancet.

[24] Moro P, Schantz PM (2009). Echinococcosis: a review. Int J Infect Dis.

[25] Eckert J, Deplazes P (2004). Biological, epidemiological, and clinical aspects of echinococcosis, a zoonosis of increasing concern. Clin Microbiol Rev.

[26] Budke CM, Deplazes P, Torgerson PR. Global socioeconomic impact of cystic echinococcosis. Emerg. Infect. Dis. 2006;12(2):296–303. 10.3201/eid1202.050499 PMC3373106 16494758

[27] World Health Organization and World Organisation for Animal Health. Report of the WHO informal working group on cystic and alveolar echinococcosis surveillance, prevention and control, with the participation of the Food and Agriculture Organization of the United Nations and the World Organisation for Animal Health. 2011. http://apps.who.int/iris/bitstream/10665/44785/1/9789241502924_eng.pdf. Accessed 15 December 2014.

[28] Torgerson PR, Keller K, Magnotta M, Ragland N (2010). The global burden of alveolar echinococcosis. PLoS Negl Trop Dis.

[29] Laurimaa L, Davison J, Süld K, Plumer L, Oja R, Moks E (2015). First report of highly pathogenic Echinococcus granulosus genotype G1 in dogs in a European urban environment. Parasites Vectors.

[30] Boufana B, Lett WS, Lahmar S, Buishi I, Bodell AJ, Varcasia A (2015). Echinococcus equinus and Echinococcus granulosus sensu stricto from the United Kingdom: genetic diversity and haplotypic variation. Int J Parasitol.

[31] Schweiger A, Ammann RW, Candinas D, Clavien P-A, Eckert J, Gottstein B (2007). Human Alveolar Echinococcosis after Fox Population Increase. Switzerland Emerg. Infect. Dis..

[32] Usubalieva J, Minbaeva G, Ziadinov I, Deplazes P, Torgerson PR (2013). Human alveolar echinococcosis in Kyrgyzstan. Emerg Infect Dis.

[33] Lind EO, Juremalm M, Christensson D, Widgren S, Hallgren G, Ågren E (2011). First detection of Echinococcus multilocularis in Sweden, February to March 2011. Euro Surveill.

[34] Jenkins EJ, Peregrine AS, Hill JE, Somers C, Gesy K, Barnes B (2012). Detection of European strain of Echinococcus multilocularis in North America. Emerg Infect Dis.

[35] Yang D, Zhang T, Zeng Z, Zhao W, Zhang W, Liu A (2015). The first report of human-derived G10 genotype of Echinococcus canadensis in China and possible sources and routes of transmission. Parasitol Int.

[36] Tappe D, Stich A, Frosch M (2008). Emergence of polycystic neotropical echinococcosis. Emerg Infect Dis.

[37] Gilot B, Doche B, Deblock S, Petavy A (1988). Ecological Cartography of Alveolar Echinococcosis in the Central Massif (France)-Attempt to delimit a center of infection Canadian. J Zoology-Revue Canadienne De Zoologie.

[38] Giraudoux P, Vuitton D, Bresson-Hadni S, Craig P, Bartholomot B, Barnish G (1996). Mass screening and epidemiology of Alveolar echinococcosis in France, Western Europe, and in Gansu, Central China: from epidemiology towards transmission ecology.

[39] Viel J-F, Giraudoux P, Abrial V, Bresson-Hadni S (1999). Water vole (Arvicola terrestris scherman) density as risk factor for human alveolar echinococcosis. AmJTrop Med Hyg.

[40] Berke O (2001). Choropleth mapping of regional count data of Echinococcus multilocularis among red foxes in Lower Saxony, Germany. Prev. Vet. Med..

[41] Giraudoux P, Craig P, Delattre P, Bao G, Bartholomot B, Harraga S (2003). Interactions between landscape changes and host communities can regulate Echinococcus multilocularis transmission. Parasitology.

[42] Graham AJ, Danson FM, Craig PS (2005). Ecological epidemiology: the role of landscape structure in the transmission risk of the fox tapeworm Echinococcus multilocularis (Leukart 1863) (Cestoda : Cyclophyllidea : Taeniidae). Prog Phys Geogr.

[43] Pleydell DR, Yang YR, Danson FM, Raoul F, Craig PS, McManus DP (2008). Landscape composition and spatial prediction of alveolar echinococcosis in southern Ningxia, China. PLoS Negl Trop Dis.

[44] Atkinson J-AM, Gray DJ, Clements ACA, Barnes TS, McManus DP, Yang YR (2013). Environmental changes impacting Echinococcus transmission: research to support predictive surveillance and control. Glob Chang Biol.

[45] Danson F, Graham A, Pleydell D, Campos-Ponce M, Giraudoux P, Craig P (2003). Multi-scale spatial analysis of human alveolar echinococcosis risk in China. Parasitology.

[46] Veit P, Bilger B, Schad V, Schäfer J, Frank W, Lucius R (1995). Influence of environmental factors on the infectivity of Echinococcus multilocularis eggs. Parasitology.

[47] Thevenet PS, Jensen O, Drut R, Cerrone GE, Grenóvero MS, Alvarez HM (2005). Viability and infectiousness of eggs of Echinococcus granulosus aged under natural conditions of inferior arid climate. Vet Parasitol.

[48] Macpherson C (1983). An active intermediate host role for man in the life cycle of Echinococcus granulosus in Turkana, Kenya. AmJTrop Med Hyg.

[49] Otero-Abad B, Torgerson PR (2013). A systematic review of the epidemiology of echinococcosis in domestic and wild animals.

[50] Giraudoux P, Pleydell D, Raoul F, Vaniscotte A, Ito A, Craig PS (2007). Echinococcus Multilocularis: Why are multidisciplinary and multiscale approaches essential in infectious disease ecology?. Tropical Med Health.

[51] Giraudoux P, Pleydell D, Raoul F, Quéré J-P, Wang Q, Yang Y (2006). Transmission ecology of Echinococcus multilocularis: What are the ranges of parasite stability among various host communities in China?. Parasitol Int.

[52] Giraudoux P, Raoul F, Afonso EVE, Ziadinov I, Yang Y, Li LI (2013). Transmission ecosystems of Echinococcus multilocularis in China and Central Asia. Parasitology.

[53] Tolnai Z, Széll Z, Sréter T (2013). Environmental determinants of the spatial distribution of Echinococcus multilocularis in Hungary. Vet Parasitol.

[54] Wang Q, Vuitton DA, Qiu J, Giraudoux P, Xiao Y, Schantz PM (2004). Fenced pasture: a possible risk factor for human alveolar echinococcosis in Tibetan pastoralist communities of Sichuan. China Acta Tropica.

[55] Wang Q, Vuitton DA, Xiao Y, Budke CM, Campos-Ponce M, Schantz PM (2006). Pasture types and Echinococcus multilocularis, Tibetan communities. Emerg Infect Dis.

[56] Giraudoux P, Delattre P, Habert M, Quéré JP, Deblay S, Defaut R (1997). Population dynamics of fossorial water vole (Arvicola terrestris scherman): a land use and landscape perspective. Agric Ecosyst Environ.

[57] Raoul F, Deplazes P, Nonaka N, Piarroux R, Vuitton DA, Giraudoux P (2001). Assessment of the epidemiological status of Echinococcus multilocularis in foxes in France using ELISA coprotests on fox faeces collected in the field. Int J Parasitol.

[58] Craig P, Giraudoux P, Shi D, Bartholomot B, Barnish G, Delattre P (2000). An epidemiological and ecological study of human alveolar echinococcosis transmission in south Gansu, China. Acta Trop.

[59] Wang Q, Xiao Y-f, Vuitton DA, Schantz PM, Raoul F, Budke C (2007). Impact of overgrazing on the transmission of Echinococcus multilocularis in Tibetan pastoral communities of Sichuan Province, China. Chinese Med J-Beijing-English Edition.

[60] Wang Q, Raoul F, Budke C, Craig PS, Xiao Y-f, Vuitton DA (2010). Grass height and transmission ecology of Echinococcus multilocularis in Tibetan communities, China. Chinese Med J (English Edition).

[61] Romig T, Thoma D, Weible A-K (2006). Echinococcus multilocularis–a zoonosis of anthropogenic environments?. J Helminthol.

[62] Saitoh T, Stenseth NC, Bjørnstad ON (1998). The population dynamics of the vole Clethrionomys rufocanus in Hokkaido, Japan. Res Popul Ecol.

[63] Marston CG, Danson FM, Armitage RP, Giraudoux P, Pleydell DR, Wang Q (2014). A random forest approach for predicting the presence of Echinococcus multilocularis intermediate host Ochotona spp. presence in relation to landscape characteristics in western China. Appl Geogr.

[64] Thornton PK, van de Steeg J, Notenbaert A, Herrero M (2009). The impacts of climate change on livestock and livestock systems in developing countries: A review of what we know and what we need to know. Agric Syst.

[65] Sevi A, Caroprese M (2012). Impact of heat stress on milk production, immunity and udder health in sheep: A critical review. Small Rumin Res.

[66] Ansari-Lari M (2005). A retrospective survey of hydatidosis in livestock in Shiraz, Iran, based on abattoir data during 1999–2004. Vet Parasitol.

[67] Ibrahim MM (2010). Study of cystic echinococcosis in slaughtered animals in Al Baha region, Saudi Arabia: Interaction between some biotic and abiotic factors. Acta Trop.

[68] Acosta-Jamett G, Cleaveland S, Cunningham AA, Barend M, Craig PS (2010). Echinococcus granulosus infection in humans and livestock in the Coquimbo region, north-central Chile. Vet Parasitol.

[69] Fromsa A, Jobre Y. Infection prevalence of hydatidosis (Echinococcus granulosus, Batsch, 1786) in domestic animals in Ethiopia: A synthesis report of previous surveys. Ethiopian veterinary journal. 2011;15(2):11–33.

[70] Cringoli G, Rinaldi L, Musella V, Veneziano V, Maurelli MP, Di Pietro F (2007). Geo-referencing livestock farms as tool for studying cystic echinococcosis epidemiology in cattle and water buffaloes from southern Italy. Geospatial Health.

[71] Federer K, Armua-Fernandez MT, Hoby S, Wenker C, Deplazes P (2015). In vivo viability of Echinococcus multilocularis eggs in a rodent model after different thermo-treatments. Exp Parasitol.

[72] Fox NJ, Marion G, Davidson RS, White PC, Hutchings MR (2012). Livestock helminths in a changing climate: approaches and restrictions to meaningful predictions. Animals.

[73] Deplazes P, Hegglin D, Gloor S, Romig T (2004). Wilderness in the city: the urbanization of *Echinococcus multilocularis*. Trends Parasitol.

[74] Liccioli S, Giraudoux P, Deplazes P, Massolo A (2015). Wilderness in the ‘city’ revisited: different urbes shape transmission of Echinococcus multilocularis by altering predator and prey communities. Trends Parasitol.

[75] Giraudoux P, Raoul F, Pleydell D, Li T, Han X, Qiu J (2013). Drivers of echinococcus multilocularis transmission in China: small mammal diversity, landscape or climate?. PLoS Negl Trop Dis.

[76] Moss J, Chen X, Li T, Qiu J, Wang Q, Giraudoux P (2013). Reinfection studies of canine echinococcosis and role of dogs in transmission of Echinococcus multilocularis in Tibetan communities, Sichuan. China Parasitol.

[77] Ziadinov I, Mathis A, Trachsel D, Rysmukhambetova A, Abdyjaparov TA, Kuttubaev OT (2008). Canine echinococcosis in Kyrgyzstan: Using prevalence data adjusted for measurement error to develop transmission dynamics models. Int J Parasitol.

[78] Liccioli S, Bialowas C, Ruckstuhl KE, Massolo A (2015). Feeding Ecology Informs Parasite Epidemiology: Prey Selection Modulates Encounter Rate with Echinococcus multilocularis in Urban Coyotes. PLoS One.

[79] Pleydell DJ, Raoul F, Vaniscotte A, Craig P, Giraudoux P, Morand S, Krasnov B, Poulin R (2006). Towards understanding the impacts of environmental variation on Echinococcus multilocularis transmission. Micromammals and Macroparasites.

[80] Vuitton DA, Gottstein B (2010). Echinococcus multilocularis and its intermediate host: a model of parasite-host interplay. Bio Med Res Int.

[81] Chauchet A, Grenouillet F, Knapp J, Richou C, Delabrousse E, Dentan C (2014). Increased incidence and characteristics of alveolar echinococcosis in patients with immunosuppression-associated conditions. Clin Infect Dis.

[82] Koch S, Bresson-Hadni S, Miguet J-P, Crumbach J-P, Gillet M, Mantion G-A (2003). Experience of liver transplantation for incurable alveolar echinococcosis: a 45-case European collaborative report. Transplantation.

[83] Kern P, Grüner B, Wahlers K (2011). Diagnosis and course of echinococcocal diseases in the transplant setting. Transpl Infect Dis.

[84] Danson FM, Giraudoux P, Craig PS (2006). Spatial modelling and ecology of Echinococcus multilocularis transmission in China. Parasitol Int.

[85] Tiaoying L, Jiamin Q, Wen Y, Craig PS, Xingwang C, Ning X (2005). Echinococcosis in Tibetan populations, western Sichuan province, China. Emerg Infect Dis.

[86] Chi P, Zhang W, Zhang Z, Hasyet M, Liu F, Ding Z (1990). Cystic echinococcosis in the Xinjiang/Uygur Autonomous Region, People’s Republic of China. I. Demographic and epidemiologic data. Trop. Med. Parasitol..

[87] Cohen H, Paolillo E, Bonifacino R, Botta B, Parada L, Cabrera P (1998). Human cystic echinococcosis in a Uruguayan community: a sonographic, serologic, and epidemiologic study. AmJTrop Med Hyg.

[88] Kern P, Bardonnet K, Renner E, Auer H, Pawlowski Z, Ammann RW (2003). European echinococcosis registry: human alveolar echinococcosis, Europe, 1982–2000. Emerg Infect Dis.

[89] Caprarelli G, Fletcher S (2014). A brief review of spatial analysis concepts and tools used for mapping, containment and risk modelling of infectious diseases and other illnesses. Parasitology.

[90] Bartholomot G, Vuitton DA, Harraga S, Shi DZ, Giraudoux P, Barnish G (2002). Combined ultrasound and serologic screening for hepatic alveolar echinococcosis in central China. AmJTrop Med Hyg.

[91] Yang Y, Williams G, Craig P, Sun T, Yang S, Cheng L (2006). Hospital and community surveys reveal the severe public health problem and socio‐economic impact of human echinococcosis in Ningxia Hui Autonomous Region. China Trop. Med. Int. Health.

[92] Kilimcioğlu AA, Girginkardeşler N, Korkmaz M, Özkol M, Düzgün F, Östan İ (2013). A mass screening survey of cystic echinococcosis by ultrasonography, Western blotting, and ELISA among university students in Manisa. Turkey Acta Tropica.

[93] Bingham GM, Budke CM, Larrieu E, Del Carpio M, Mujica G, Slater MR (2014). A community-based study to examine the epidemiology of human cystic echinococcosis in Rio Negro Province. Argentina Acta Tropica.

[94] Charbonnier A, Knapp J, Demonmerot F, Bresson-Hadni S, Raoul F, Grenouillet F et al. A new data management system for the French National Registry of human alveolar echinococcosis cases. Parasite. 2014;21:69. 10.1051/parasite/2014075 PMC4271653 25526544

[95] Said-Ali Z, Grenouillet F, Knapp J, Bresson-Hadni S, Vuitton DA, Raoul F (2013). Detecting nested clusters of human alveolar echinococcosis. Parasitology.

[96] Schneider R, Aspöck H, Auer H (2013). Unexpected increase of alveolar echincoccosis, Austria, 2011. Emerg Infect Dis.

[97] Melesse AM, Weng Q, Thenkabail PS, Senay GB (2007). Remote sensing sensors and applications in environmental resources mapping and modelling. Sensors.

[98] Samanta S, Pal D, Lohar D, Pal B (2012). Interpolation of climate variables and temperature modeling. Theor Appl Climatol.

[99] Schabenberger O, Gotway CA. Statistical methods for spatial data analysis. CRC press; 2004.

[100] The International Union for Conservation of Nature. The IUCN Red List of Threatened Species. http://www.iucnredlist.org/. Accessed 7 September 2015.

[101] Piarroux M, Piarroux R, Knapp J, Bardonnet K, Dumortier J, Watelet J (2013). Populations at risk for alveolar echinococcosis, France. Emerg Infect Dis.

[102] Cassini R, Mulatti P, Zanardello C, Simonato G, Signorini M, Cazzin S (2014). Retrospective and spatial analysis tools for integrated surveillance of cystic echinococcosis and bovine cysticercosis in hypo-endemic areas. Geospatial Health.

[103] Manfredi M, Cerbo A, Zanzani S (2013). Cystic echinococcosis in Lombardy: epidemiological aspects and spatial analysis. Helminthologia.

[104] Takahashi K, Uraguchi K, Kudo S (2005). The epidemiological status of Echinococcus multilocularis in animals in Hokkaido, Japan. Mammal Study.

[105] Li Y, Yan J, Li J, Zhang L, Zhang L, Jang B (2005). Investigation and analysis of epidemic conditions of hydatid disease of sheep in Xinjiang. J Shihezi Univ (Natural Science).

[106] Craig PS, Li T, Qiu J, Zhen R, Wang Q, Giraudoux P (2008). Echinococcoses and Tibetan communities. Emerg Infect Dis.

[107] Wang Q, Qiu J, Yang W, Schantz PM, Raoul F, Craig PS (2006). Socioeconomic and behavior risk factors of human alveolar echinococcosis in Tibetan communities in Sichuan, People’s Republic of China. AmJTrop Med Hyg.

[108] Yang YR, Sun T, Li Z, Zhang J, Teng J, Liu X (2006). Community surveys and risk factor analysis of human alveolar and cystic echinococcosis in Ningxia Hui Autonomous Region, China. Bull World Health Organ.

[109] Acosta-Jamett G, Weitzel T, Boufana B, Adones C, Bahamonde A, Abarca K (2014). Prevalence and risk factors for echinococcal infection in a rural area of northern Chile: a household-based cross-sectional study.

[110] Danson FM, Craig PS, Man W, Shi D, Giraudoux P (2004). Landscape dynamics and risk modeling of human alveolar echinococcosis. Photogramm Eng Remote Sens.

[111] Elliott P, Wartenberg D. Spatial epidemiology: current approaches and future challenges. Environ. Health Perspect. 2004;112(9):998–1006. 10.1289/ehp.6735 PMC1247193 15198920

[112] Clyde M, George EI. Model uncertainty. Stat Sci. 2004:81–94.

[113] Giardina F, Kasasa S, Sié A, Utzinger J, Tanner M, Vounatsou P (2014). Effects of vector-control interventions on changes in risk of malaria parasitaemia in sub-Saharan Africa: a spatial and temporal analysis. Lancet Global Health.

[114] London School of Hygiene and Tropical Medicine. Global Atlas of Helminth Infections (GAHI). http://www.thiswormyworld.org/. Accessed 8 July 2014.

[115] Brooker S, Hotez PJ, Bundy DA (2010). The global atlas of helminth infection: mapping the way forward in neglected tropical disease control. PLoS Negl Trop Dis.

[116] Gething PW, Patil AP, Smith DL, Guerra CA, Elyazar I, Johnston GL (2011). A new world malaria map: Plasmodium falciparum endemicity in 2010. Malar J.

[117] Yang YR, Clements AC, Gray DJ, Atkinson J-AM, Williams GM, Barnes TS (2012). Impact of anthropogenic and natural environmental changes on Echinococcus transmission in Ningxia Hui Autonomous Region, the People’s Republic of China. Parasites Vectors.

[118] Zhang W, Zhang Z, Wu W, Shi B, Li J, Zhou X et al. Epidemiology and control of echinococcosis in central Asia, with particular reference to the People’s Republic of China. Acta Tropica. 2015;141, Part B(0):235–43. 10.1016/j.actatropica.2014.03.014 24686096

[119] Ikeda T, Yoshimura M, Onoyama K, Oku Y, Nonaka N, Katakura K (2014). Where to deliver baits for deworming urban red foxes for Echinococcus multilocularis control: new protocol for micro-habitat modeling of fox denning requirements. Parasites Vectors.

[120] Ishida A, Takahashi K, Uraguchi K, Oshida T (2014). Environmental factors for efficiently baiting red foxes in agricultural areas in eastern Hokkaido. Japan Mammal Study.

[121] Takahashi K, Uraguchi K, Hatakeyama H, Giraudoux P, Romig T (2013). Efficacy of anthelmintic baiting of foxes against Echinococcus multilocularis in northern Japan. Vet Parasitol.

[122] Comte S, Raton V, Raoul F, Hegglin D, Giraudoux P, Deplazes P (2013). Fox baiting against Echinococcus multilocularis: Contrasted achievements among two medium size cities. Prev. Vet. Med..

[123] Gething PW, Noor AM, Gikandi PW, Ogara EA, Hay SI, Nixon MS (2006). Improving imperfect data from health management information systems in Africa using space-time geostatistics. PLoS Med.

[124] Torgerson PR (2013). One world health: Socioeconomic burden and parasitic disease control priorities. Vet Parasitol.

[125] Nakagawa J, Ehrenberg JP, Nealon J, Fürst T, Aratchige P, Gonzales G (2015). Towards effective prevention and control of helminth neglected tropical diseases in the Western Pacific Region through multi-disease and multi-sectoral interventions. Acta Trop.

