# Supplementary material for: The landscape epidemiology of echinococcoses
Source: Infect Dis Poverty. 2016 Feb 19;5:13. doi: 10.1186/s40249-016-0109-x (PMC4759770; doi:10.1186/s40249-016-0109-x)
Supplement: Additional file 1: — Multilingual abstracts in the six official working languages of the United Nations. (PDF 336 kb) [file 40249_2016_109_MOESM1_ESM.pdf]

Translation of the abstract into the six official working languages of the United Nations

مشهد مواقع الانتشار الوبائي لداء الأكياس المائية

Angela M. Cadavid Restrepo, Yu Rong Yang, Donald P. McManus, Darren J. Gray, Patrick Giraudoux, Tamsin S. Barnes, Gail M. Williams, Ricardo J. Soares Magalhães, Nicholas A.S. Hamm, Archie C.A. Clements

ملخص

يعتبر مرض الأكياس المائية أحد الأمراض الطفيلية التي يجب علاجها للحفاظ على الصحة العامة على مستوى العالم. تؤدي العدوى بين البشر إلى ظهور أمراض مزمنة يصعب التكهن بتأثيرها على جسم الإنسان، وإلى ظهور عواقب طبية واجتماعية واقتصادية خطيرة على المجموعات السكانية المعرضة لهذا المرض. وفقًا لتقديرات أخيرة، فإن التوزيع الجغرافي للالتهابات التي تسببها البكتيريا من جنس الديدان المُشْوِكة تتوسع وتصبح مشكلة متكررة الحدوث في عدة أقاليم في العالم. إن استيطان داء الأكياس المائية متباين من الناحية الجغرافية ويمكن أن يتأثر بالتغيرات الطارئة على النظام البيئي العالمي مع مرور الوقت. ولهذا السبب، يتيح مشهد مواقع انتشار الأوبئة فرصة فريدة لتوقع وتحديد مقدار المخاطر البيئية للعدوى على نطاقات مكانية وزمانية متعددة. وهنا، نستعرض المصادر البيئية الأكثر أهمية لهذا التباين المكاني المتعلق بخطر إصابة الإنسان بهذا الداء ونقدم وصفًا للتطبيقات المحتملة لدراسات مشاهد مواقع انتشار الأوبئة وبالتالي تحديد خصائص الأنماط الحالية لطرق الانتقال الطفيلي عبر المواقع الطبيعية والمواقع التي يغيرها الإنسان. إننا نؤيد الجهود المستقبلية التي تسعى إلى تعزيز استخدام هذا النهج باعتباره أداة تدعم اتخاذ القرار الذي يسهل تصميم وتنفيذ ومراقبة التدخلات المكانية المستهدفة من أجل الحد من عبء داء الأكياس المائية على الإنسان في المناطق الموبوءة.

Translated from English version into Arabic by Mahmoud Sami, through

TRANSLATORS
WITHOUT BORDERS

包虫病与地理环境相关的流行病学

Angela M. Cadavid Restrepo, Yu Rong Yang, Donald P. McManus, Darren J. Gray, Patrick Giraudoux, Tamsin S. Barnes, Gail M. Williams, Ricardo J. Soares Magalhães, Nicholas A.S. Hamm, Archie C.A. Clements

摘要

包虫病是严重威胁全球公共卫生的主要寄生虫病。人类感染能导致预后差的慢性病程，给弱势群体造成严重的医疗，社会和经济负担后果。根据最新资料估计，两种棘球属绦虫的传播和感染有扩大地理分布的趋势，也即在世界范围不断有新发现的流行地域，和一些曾经被控制的地区重新出现流行的问题。包虫病流行在地理分布上的不均衡性，和随着时间推移可能会受全球环境变化的影响而发生的分布变化。因此，地理环境流行病学提供了一个非常有用的工具和机会，能够在多重空间和时间跨度下量化和预测传播和感染的生态风险。在此综述中，我们回顾性的研究了环境资源和空间变化对人类包虫病传播相关的风险，并描述了地理景观流行病学的应用研究程序能够揭示在当前自然和人为改变的地理环境条件下，寄生虫传播潜在的模式和特征。我们提倡今后的工作中推广使用这种方法作为辅助工具，有利于进行决策和针对性的空间干预措施的设计，实施和监督，以减少疾病流行区人患包虫病的疾病负担。

Translated from English version into Chinese by Yu Rong Yang

L'épidémiologie environnementale des échinococcoses

Angela M. Cadavid Restrepo, Yu Rong Yang, Donald P. McManus, Darren J. Gray, Patrick Giraudoux, Tamsin S. Barnes, Gail M. Williams, Ricardo J. Soares Magalhães, Nicholas A.S. Hamm, Archie C.A. Clements

Résumé

Les échinococcoses sont des maladies parasitaires d'une importance majeure en termes de santé publique au niveau mondial. L'infection humaine entraîne une maladie chronique, au pronostic défavorable et aux conséquences médicales, sociales et économiques graves pour les populations vulnérables. Des estimations récentes indiquent que la répartition géographique des infections causées par les *Echinococcus* spp. progresse pour devenir un problème émergent et résurgent dans plusieurs régions du monde. Si l'endémie de l'échinococcose est géographiquement hétérogène, elle pourrait, avec le temps, être influencée par le changement écologique mondial. C'est pourquoi l'épidémiologie environnementale présente une opportunité unique de quantifier et de prévoir le risque écologique d'infection à de multiples échelles, spatiales et temporelles. Nous passons en revue les sources écologiques les plus pertinentes en matière de variation spatiale du risque humain d'échinococcose, puis décrivons les applications potentielles des études d'épidémiologie environnementale en vue de caractériser les modes actuels de transmission du parasite au sein des zones géographiques naturelles et altérées par l'homme. À l'avenir, nous préconisons de poursuivre ce travail, qui devra promouvoir l'utilisation de cette approche en tant qu'outil de soutien à la prise de décisions facilitant la conception, la mise en œuvre et le contrôle d'interventions géographiquement ciblées visant à réduire le poids des échinococcoses humaines dans les zones de maladies endémiques.

Translated from English version into French by Clèmentine Choubrac

Ландшафтная эпидемиология эхинококкоза

Анджела М. Кандавид Рестрепо, Ю Жонг Янг, Дональд П. Макманус, Даррен Дж. Грей, Патрик Жирадо, Тамсин С. Беймс, Гейл М. Вильямс, Рикардо Х. Соарес Магальяес, Николас А.С. Хамм, Арчи С.А. Клементс

Резюме

Эхинококкоз – паразитическое заболевание, которое имеет большое значение для здоровья населения всего земного шара. Заражение человека эхинококком приводит к хроническому заболеванию с неблагоприятным прогнозом и серьезными медицинскими, социальными и экономическими последствиями для уязвимых групп населения. По последним оценкам, география распространения заражения различными видами *Echinococcus* расширяется и является новой и рецидивирующей проблемой в ряде регионов мира. Эндемичность эхинококкоза неоднородна в географическом плане и со временем на нее может повлиять глобальное изменение окружающей среды. Поэтому ландшафтная эпидемиология предлагает уникальную возможность количественно измерять и прогнозировать экологический риск заражения в различных пространственных и временных масштабах. Здесь мы рассматриваем наиболее важные природные источники пространственного изменения степени риска человеческого эхинококкоза и описываем возможности применения ландшафтных эпидемиологических исследований для характеристики современных схем распространения паразитов в естественных и антропогенных ландшафтах. Мы

продвигаем направления дальнейших исследований с использованием данного метода в качестве вспомогательного средства принятия решений, облегчающего разработку, внедрение и мониторинг территориально направленных мер по снижению бремени человеческого эхинококкоза в эндемических областях.

Translated from English version into Russian by Jekaterina Merkuljeva, through

La epidemiología paisajística de la equinococosis

Angela M. Cadavid Restrepo, Yu Rong Yang, Donald P. McManus, Darren J. Gray, Patrick Giraudoux, Tamsin S. Barnes, Gail M. Williams, Ricardo J. Soares Magalhães, Nicholas A.S. Hamm, Archie C.A. Clements

Resumen
Las equinococosis son enfermedades parasitarias de significativa importancia para la salud pública a nivel mundial. La infección en el ser humano resulta en una enfermedad crónica de mal pronóstico y serias consecuencias médicas, sociales y económicas para aquellas poblaciones vulnerables. De acuerdo a estimaciones recientes, la distribución geográfica de las infecciones por *Echinococcus* spp. se están propagando y se están convirtiendo en un problema emergente y re-emergente en varias regiones del mundo. La endemicidad de la equinococosis es geográficamente heterogénea y con el tiempo puede haber sido afectada por el cambio ambiental global. Por lo tanto, la epidemiología paisajística ofrece una oportunidad única de cuantificar y predecir el riesgo ecológico de la infección en múltiples escalas espaciales y temporales. Aquí, analizamos las fuentes ambientales de variación espacial más relevantes en el riesgo de la equinococosis humana y describimos las posibles aplicaciones de los estudios de epidemiología paisajística para caracterizar los patrones actuales de transmisión de parásitos en los paisajes naturales y alterados por el hombre. Proponemos trabajos futuros que promocionen el uso de este método como herramienta de soporte para la toma de decisiones que faciliten el diseño, la implementación y el monitoreo de intervenciones espaciales para reducir la carga de la equinococosis humana en las áreas endémicas para la enfermedad.

Translated from English version into Spanish by Maria Alejandra Aguada, through
